# Spatiotemporal Symmetries and Energy-Momentum Conservation in Uniform Spacetime Metamaterials

**DOI:** 10.1021/acsphotonics.4c01496

**Published:** 2024-11-13

**Authors:** Iñigo Liberal, Antonio Ganfornina-Andrades, J. Enrique Vázquez-Lozano

**Affiliations:** Department of Electrical, Electronic and Communications Engineering, Institute of Smart Cities (ISC), Public University of Navarre (UPNA), 31006 Pamplona, Spain

**Keywords:** spatiotemporal metamaterials, spacetime metamaterials, time-varying media, Noether’s theorem, symmetries

## Abstract

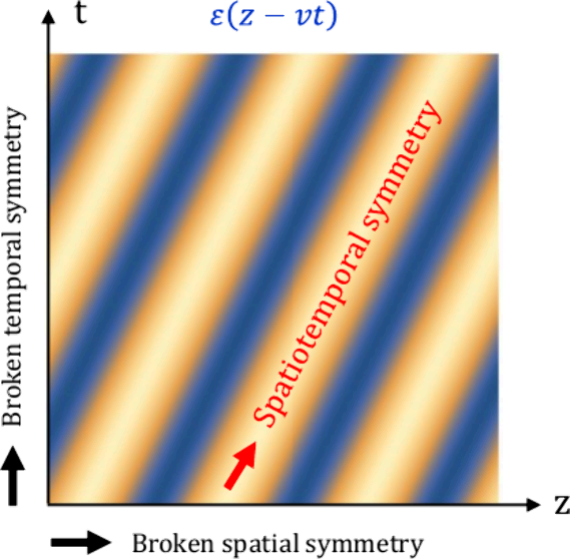

Spacetime metamaterials are opening new regimes of light–matter interactions based on the breaking of temporal and spatial symmetries, as well as intriguing concepts associated with synthetic motion. In this work, we investigate the continuous spatiotemporal translation symmetry of spacetime metamaterials with uniform modulation velocity. Using Noether’s theorem, we demonstrate that such symmetry entails the conservation of the energy momentum. We highlight how energy-momentum conservation imposes constraints on the range of allowed light–matter interactions within spacetime metamaterials, as illustrated with examples of the collision of electromagnetic and modulation pulses. Furthermore, we discuss the similarities and differences between the conservation of energy-momentum and relativistic effects. We believe that our work provides a step forward in clarifying the fundamental theory underlying spacetime metamaterials.

## 1 Introduction

Recent experimental efforts on the ultrafast optical modulation of the refractive index of materials,^1−6^ as well as the electronic control of metamaterial waveguides^7−9^ and metasurfaces,^10^ are opening the pathway toward new regimes of light-matter interactions. In essence, the possibility of modulating the properties of optical systems at time-scales comparable to the frequency of operation have opened a shared multidisciplinary field that currently lies under the names of four-dimensional (4D) optics,^11^ time-varying media (TVM)^12^ and/or spacetime metamaterials (ST-MMs).^13,14^

As it is usual in physics, much of the new phenomena can be traced back to the breaking of symmetries that are conventionally preserved. Noether’s theorem^15,16^ states that any continuous symmetry is associated with a conserved quantity and, therefore, breaking a symmetry removes the constraints imposed by the conservation of such quantity. For example, breaking the continuous temporal translation symmetry lifts the restrictions imposed by energy conservation, which enables novel forms of amplification and light emission. Examples include modified quantum vacuum amplification effects,^17,18^ thermal emission,^19^ frequency shifting with quantum noise management,^20^ as well as emission from localized quantum emitters^21^ and free-electrons.^22^ Similarly, breaking time-reversal symmetry lifts the restrictions imposed by reciprocity, which has enabled new technologies of magnet-less nonreciprocal devices.^23,24^

Despite the interest of the broken symmetries, an equally relevant question is what symmetries remain intact, and what are the limitations imposed by them. For example, a purely time-modulated metamaterial preserves spatial translation symmetries. Therefore, the Minkowski^25,26^ momentum of the electromagnetic field is conserved,^27^ imposing correlations between forward and backward waves. Similarly, a purely time-modulated metamaterial preserves rotational symmetries, but generally breaks duality and temporal symmetries, emphasizing the underlying differences between spin angular momentum, helicity and chirality.^28^

In this work, we investigate the spatiotemporal symmetries of uniform spacetime metamaterials (USTMs), i.e., metamaterials where the spatiotemporal modulation of the permittivity and permeability can be characterized by a uniform velocity *v*. As it will be shown, uniform spacetime metamaterials present a spatiotemporal symmetry with characteristic velocity *v*, which leads to the conservation of energy-momentum relations. This condition, while less restrictive than the independent conservation of energy and momentum, imposes limitations for the light-matter interactions that can take place within a uniform spacetime metamaterial.

Uniform spacetime metamaterials are a popular class of spacetime metamaterials, which have been proposed for new nonreciprocal amplifications schemes,^29−31^ generalized gratings and metasurfaces,^32−35^ quantum light emission analogue to Hawking radiation,^36,37^ Cerenkov-like radiation,^38^ frictional forces,^39^ or the observation of the Fresnel drag,^40^ just to name a few. Recently, this class of spatiotemporal modulations was experimentally demonstrated in indium tin oxide (ITO) metasurfaces, including access to superluminal regimes.^6^ Alternatively, uniform spacetime metamaterials could be implemented with pump pulses projected onto a nonlinear waveguide, as they result in an effectively 1D system being modulated at a speed corresponding to the projection of the pulse propagation on the waveguide axis. Finally, based on experimental advances in time-modulated waveguides^7−9^ and metasurfaces,^10^ uniform spacetime metamaterials could also be implemented at microwave frequencies. Our work provides a fresh perspective into these many applications, and reveals a constraint hidden within them. Notice that, other generalizations of spacetime metamaterials, e.g., recent proposals for accelerated modulations,^41^ would not present the same constraints. Therefore, our work highlight the existence of reduced symmetries and conservation laws in spacetime metamaterials, and the need to account for them in order to fully understand the opportunities of this exciting and growing field.

## 2 Spatiotemporal Translation Symmetry

**Figure 1:**
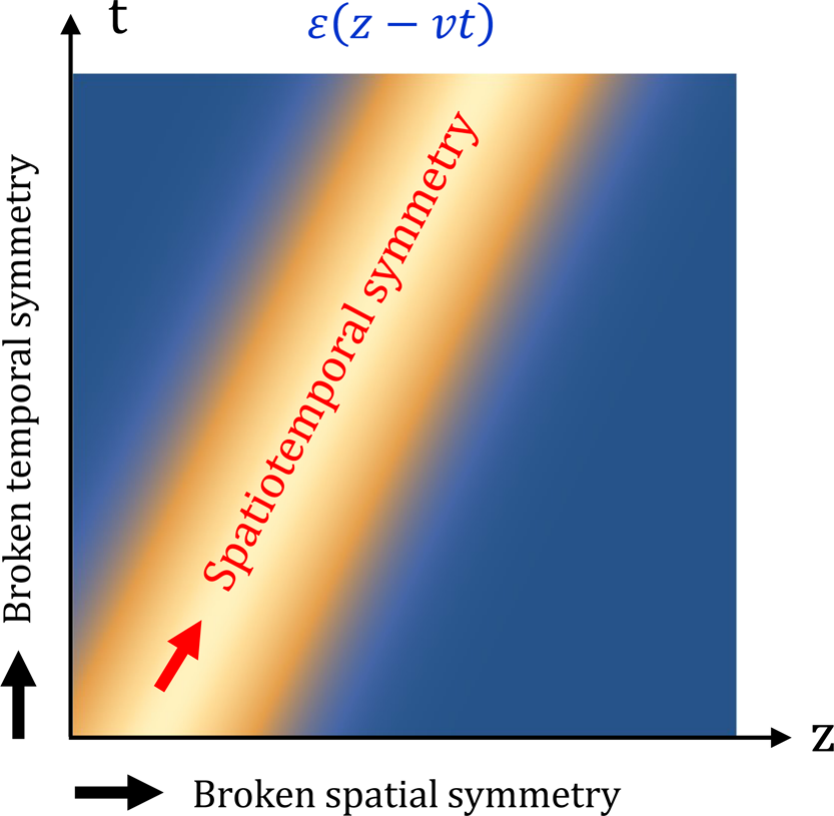
Spatiotemporal symmetries of uniform spacetime metamaterials. Schematic depiction of a uniform spacetime metamaterial where the permittivity ε(*z* – *vt*) and/or the permeability μ(*z* – *vt*) presents a spatiotemporal modulation with characteristic velocity *v*. Such system has broken symmetries along the spatial and temporal axes, but still presents a continuous translation symmetry along the *z*/*t* = *v* direction.

As schematically depicted in Figure 1, we consider a uniform spacetime metamaterials with a spatiotemporal modulation traveling along the *Z*-axis with uniform velocity *v*, characterized by permittivity ε(*z* – *vt*) permeability μ(*z* – *vt*). We work in the Coulomb gauge,^16^ so that the electric ***E***(**r**, *t*) = −∂_*t*_***A***(**r**, *t*)–∇V(**r**, *t*) and magnetic flux ***B***(**r**, *t*) = ∇ × ***A***(**r**, *t*) fields are determined by the vector ***A***(**r**, *t*) and scalar *V*(**r**, *t*) potentials, with gauge condition ∇ · ***A***(**r**, *t*). Since we are interested on the interaction with modes propagating along the direction of propagation of the modulation, without any loss of generality we consider a reduced one-dimensional problem  and, being all transversal fields, we can safely assume *V*(**r**, *t*) = 0.

In this manner, Maxwell equations reduce to the following wave equation for the vector potential:
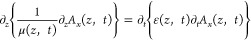
1

Then, we define a spatiotemporal continuous transformation consisting of a differential variation of the fields including temporal and spatial displacements weighted by a characteristic velocity *v*:
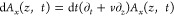
2

In passing, we note that (∂_*t*_ + *v*∂_*z*_) *A*_*x*_(*z*, *t*) = −*E*_*x*_(*z*, *t*) + *vB*_*y*_(*z*, *t*), a quantity whose continuity across a sharp spatiotemporal interfaces for moving modulations^13,14,42^ and moving matter^43^ is used as a boundary condition. Here, we remark that such quantity is not generally preserved for all times and points in space, but it represents a spatiotemporal displacement of the canonical variable *A*_*x*_(*z*, *t*), which serves for the investigation of the symmetries and globally conserved quantities of the system.

**Figure 2:**
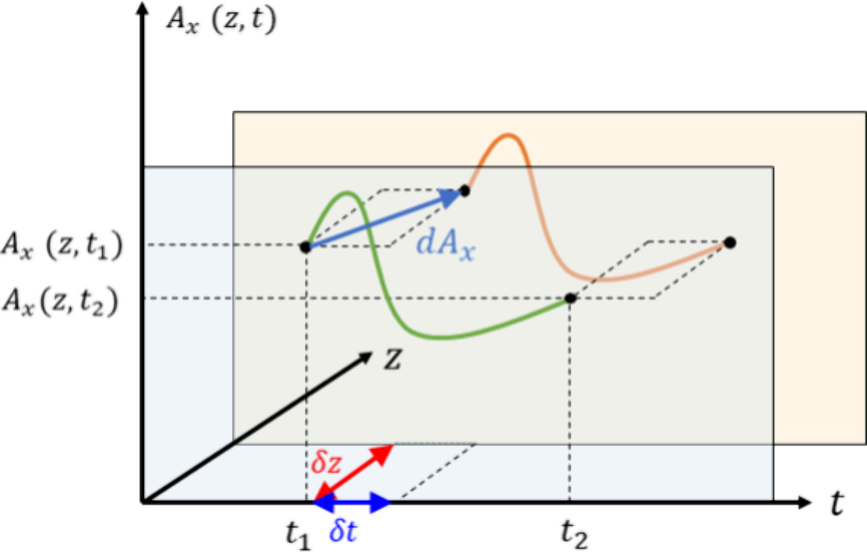
Trajectories of the vector potential under spatiotemporal translations. Schematic depiction of how dynamical variable *A*_*x*_(*z*, *t*) exhibits the same trajectory under a continuous spatiotemporal translation, identifying it as a symmetry of the system.

A continuous transformation corresponds to a symmetry when measurable outcomes are not changed by the transformation. As schematically depicted in Figure 2, it implies that the dynamical variable *A*_*x*_(*z*, *t*) and its transformed counterpart *A*′_*x*_(*z*, *t*) = *A*_*x*_(*z*, *t*) + d*A*_*x*_(*z*, *t*) will experience exactly the same trajectories. Therefore, we first check if such continuous transformation is a symmetry of Maxwell equations, i.e., if *A*′_*x*_(*z*, *t*) also obeys the wave equation given by eq 1.

As detailed in the Supporting Information Section 1, by introducing *A*′_*x*_(*z*, *t*) = *A*_*x*_(*z*, *t*) + d*A*_*x*_(*z*, *t*) into the l.h.s. of (1) and operating, the following result can be derived


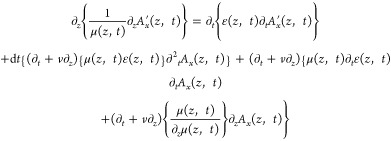
3

The existence of terms in the second, third and fourth rows of eq 3 reveal that, in general, the spatiotemporal continuous transformation (2) is not a symmetry of Maxwell equations for arbitrary spacetime modulations. However, it can be readily check that all these terms vanish provided that the permittivity and permeability modulations obey the following relations
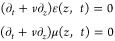
4

In other words, the continuous transformation (2) is a symmetry for a uniform spacetime metamaterial with ε(*z*, *t*) = ε(*z* – *vt*) and/or μ(*z*, *t*) = μ(*z* – *vt*). Here, it is important to remark that a homogeneous permittivity ε(*z*, *t*) = ε_cst_ and/or permeability μ(*z*, *t*) = μ_cst_ is also a uniform spatiotemporal modulation for all velocities. In other words, homogeneous media is invariant under any spatiotemporal translations, while a uniform spacetime metamaterial is only invariant for a very specific spatiotemporal translation. Therefore, the conditions given by eq 4 are satisfied in four different scenarios (i) ε(*z* – *vt*) and μ(*z* – *vt*), (ii) ε(*z* – *vt*) and μ_cst_, (iii) ε_cst_ and μ(*z* – *vt*), and (iv) ε_cst_ and μ_cst_.

## 3 Noether’s Theorem and Energy-Momentum Conservation

Next, we derive the conserved quantity associated with the spatiotemporal translation symmetry, finding that it is a linear superposition of the energy and the Minkowski momentum of the electromagnetic field. To this end, we start with a Lagrangian description of the electromagnetic field^16^, with Lagrangian density where it can be checked that Euler–Lagrange’s equation:  results in the wave eq 1.

Noether’s theorem, as previously applied to time-varying media,^27,28^ states that for any symmetry of the system, *dA*_*x*_, the following quantity must be a conserved quantity
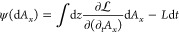
5

Accordingly, for the spatiotemporal translation symmetry given by eq 2, the conserved quantity is given by
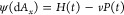
6where *H*(*t*) is the electromagnetic energy
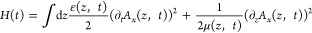
7and  is the projection of the Minkowski^25,26^ momentum of the electromagnetic field along :
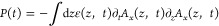
8

Therefore, the conserved quantity associated with a spatiotemporal translation is a linear superposition of energy and momentum. This point highlights that spatiotemporal metamaterials can have reduced symmetries, leading to less strict conservation laws. That is to say, energy and momentum are not required to be conserved individually, but there is a precise linear combination of them that does need to be conserved.

The coefficient of such linear superposition is given by the velocity of the modulation *v*. Consequently, the character of the energy-momentum quantity critically depends on *v*. In the *v* → 0 limit, the spatiotemporal modulation converges to a purely spatial modulation, e.g., ε(*z* – *vt*) → ε(*z*), and the energy-momentum conservation converges to the conservation of energy *H*(*t*) – *vP*(*t*) → *H*(*t*). Inversely, in the *v* → ∞ limit the energy-momentum approaches the Minkowski momentum, as it would be the case in a purely temporal modulation. Finally, energy and momentum are individually conserved in a homogeneous medium with neither spatial nor temporal modulations, as the *H*(*t*) – *vP*(*t*) must be conserved for all *v*.

## 4 Continuity Equations

A different perspective on the energy-momentum conservation can be found by deriving relevant continuity equations. To this end, we define energy *H*(*t*) = ∫d*z h*(*z*, *t*) and momentum *P*(*t*) = ∫d*z p*(*z*, *t*) densities. Then, by taking their time derivatives and rearranging the terms (see Supporting Information Section 2), we find the following continuity equations:
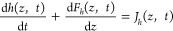
9and
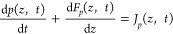
10where the energy flux is given by the Poynting vector: *F*_*h*_(*z*, *t*) = *p*(*z*, *t*)*c*^2^(*z*, *t*), while the momentum flux is given by the energy density *F*_*p*_(*z*, *t*) = *h*(*z*, *t*). In addition, the source/sink term on the r.h.s. of eq 9 is given by
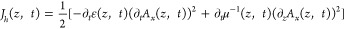
11where one intuitively confirms that the source/sinks of the energy arise from the temporal derivatives of the material parameters: ∂_*t*_ε(*z*, *t*) and ∂_*t*_μ^–1^(*z*, *t*). On the other hand, the source/sink term on the r.h.s. of eq 10 is given by
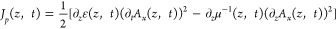
12

In this case, one intuitively confirms that the source/sinks of the momentum arise from the spatial derivatives of the material parameters: ∂_*z*_ε(*z*, *t*) and ∂_*z*_μ^–1^(*z*, *t*).

Therefore, the source/sink terms for the energy momentum density all comprise terms of the form ∂_*t*_ε(*z*, *t*) + *v*∂_*z*_ε(*z*, *t*) and ∂_*t*_μ^–1^(*z*, *t*) + *v*∂_*z*_μ^–1^(*z*, *t*) which cancel out for uniformly moving modulations ε(*z* – *vt*) and/or μ(*z* – *vt*). From this perspective, the conservation of the energy-momentum stems from the fact that the sources/sinks of energy and momentum locally cancel each other for uniformly moving modulations. Interestingly, the energy-momentum conservation can be derived from independent physical perspectives. In fact, the energy-momentum conservation predicted in this work also relates to the wavenumber-frequency locking predicted via the Fourier analysis of mirrors with spatiotemporal modulations of the reflectivity.^6^

## 5 Differences with the Four-Momentum

Spatiotemporal metamaterials are often discussed as analogues of relativity effects and/or synthetic motion.^14^ From this perspective, the structure of the conserved energy-momentum *H*(*t*) – *vP*(*t*) presents some similarities with the four-momentum of a particle  with conserved length , or the stress tensor of the electromagnetic field. The similarity arises from the mixing of energy and momentum through a preferred velocity, as well as the minus signed involved in the conservation. At the same time, it should be very clear that both conserved quantities emerge from different considerations and are fundamentally different. Examples of the differences between both quantities include: (i) The conserved value is a linear combination of energy and momentum, instead of a quadratic one. (ii) The scaling relationship between energy and momentum is given by the modulation velocity, instead of the velocity of light. (iii) For each modulation velocity, there is a different value of the conserved quantity *H* – *vP*, while  has the same value for all inertial frames, irrespectively of their associated velocity.

## 6 Consequences of the Energy-Momentum Conservation

The conservation of the energy-momentum imposes constraints on the range of light-matter interactions that can take place within the framework of uniform spacetime metamaterials. Specifically, it imposes that the changes of energy and momentum must be intertwined. To illustrate this point, we note that *H*(*t*_2_) – *vP*(*t*_2_) = *H*(*t*_1_) – *vP*(*t*_1_) can be rewritten as Δ*H*/Δ*P* = *v*, showing the explicit relation between energy and momentum variations mediated by the velocity of the modulation. A crucial factor is also the alignment of the direction of the modulation and the initial momentum of the electromagnetic field, i.e., if the electromagnetic fields and the modulation are copropagating or counterpropagating.

For example, let us consider the case in which the initial momentum and the velocity of the modulation are positive: *P*(*t*_1_) > 0 and *v* > 0. In this case, “amplifying” in the sense of Δ*H* > 0 implies that the momemtum must be increased by Δ*H*/*v*. Here, we remark that Δ*H* > 0 represents amplification in a general sense, potentially including both frequency shift and photon production processes.^20^ Furthermore, the faster the modulation the smaller the increase of momentum per unit of energy. In the *v* → ∞ limit the momentum is conserved even if the energy changes. Conversely, in the *v* → 0 limit energy is conserved, so that it does not change even if the momentum changes. However, “reflecting” in the sense of reverting the direction of the momentum of the field, i.e., *P*(*t*_2_) < 0, or, equivalently, Δ*P* < −*P*(*t*_1_), implies that the energy must be reduced by Δ*H* < −*vP*(*t*_1_). Thus, it can only be achieved if there is enough initial energy *H*(0) > *vP*(0).

On the other hand, in the counter-propagating case the initial momentum is positive *P*(*t*_1_) > 0 and the velocity of the modulation is negative *v* < 0, then Δ*H* > 0 implies that the momentum must be decreased by Δ*H*/*v*, with similar asymptotic limits. Furthermore, a decrease of the momentum is accompanied by amplification.

## 7 Example of Scattering Interactions

In this section, we utilize the theory above to analyze scattering interactions between localized electromagnetic pulses and traveling modulations. We use this scenario as a case study to illustrate different regimes for the exchange of energy and momentum, as well as the overall energy-momentum conservation. Specifically, we use a “Sech” electromagnetic pulse^44^ defined via its initial conditions at *t* = 0, before any interaction takes place: *D*_*x*_(*z*, *t* = 0) = Sech(*z*/Δ*z*) sin(2π*z*/*Z*_*p*_). We also use a traveling step modulation, i.e., ε(*z* – *vt*) = ε_b_*f*(*z* – *vt*) and μ(*z* – *vt*) = μ_b_*f*(*z* – *vt*) with *f*(*Z*) = *f*_1_ for *Z* ≤ *Z*_1_ and *Z* ≥ *Z*_2_, and *f*(*Z*) = *f*_2_ for *Z*_1_ < *Z* < *Z*_2_. This impedance-matched modulation results in a local modulation of the speed of light *c*(*z* – *vt*) = *c*_b_/*f*(*z* – *vt*) and . It simplifies the analysis while keeping relevant features of the energy-momentum conservation, and it is a common modulation within the context of USTMs.^30,36,40,45^ For such impedance-matched modulation, the partial differential equations that describe wave propagation can be solved via the method of characteristics.^46^ In particular, the displacement field can be written as follows
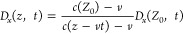
13where *Z*_0_ is the crossing the with the *T* = 0 axis of the characteristic trajectory defined by , in the transformed coordinate system *Z* = *z* – *vt* and *T* = *t*. All mathematical derivations involved are reported in Supporting Information Section 3.

**Figure 3:**
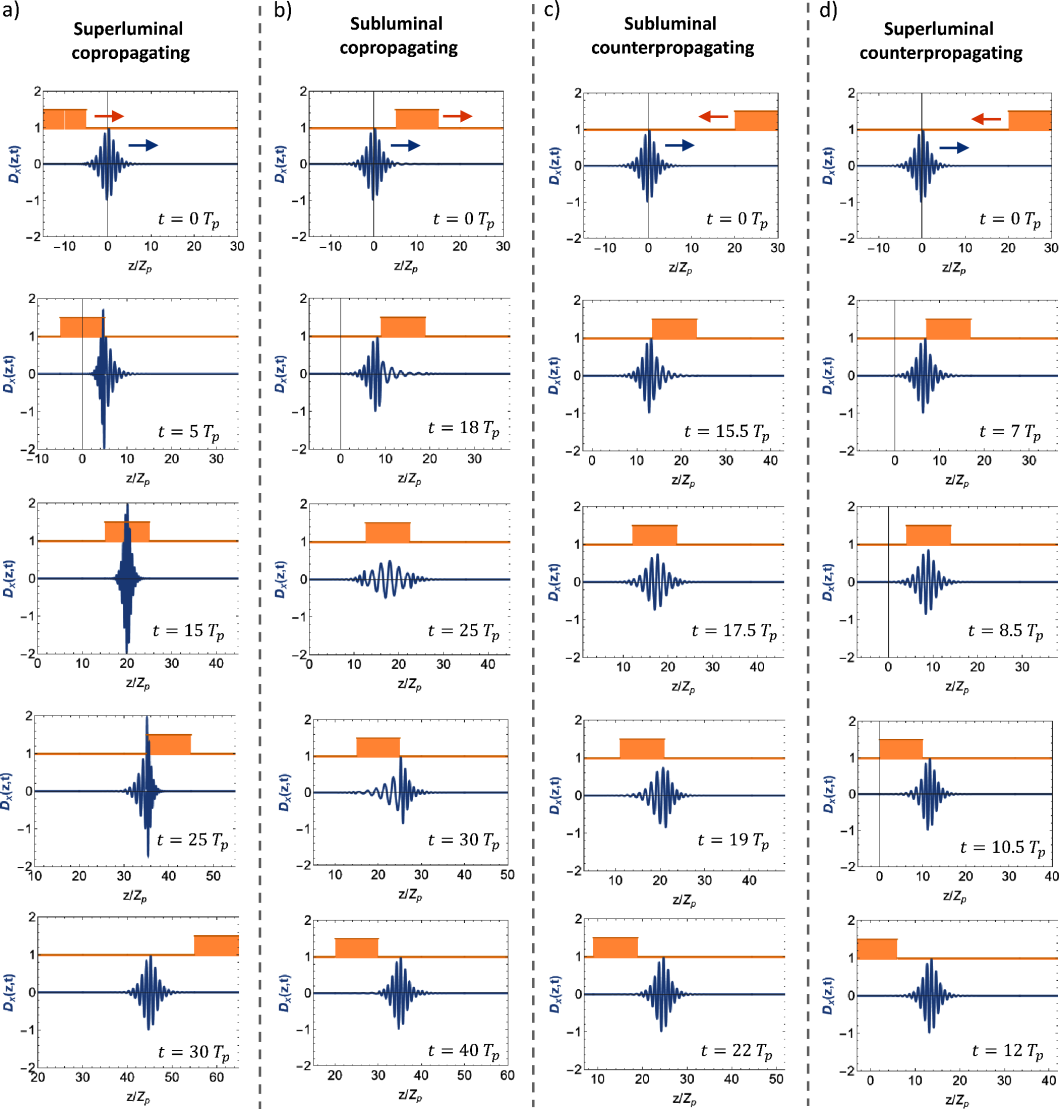
Snapshots of scattering interactions in different regimes. Displacement field *D*_*x*_(*z*, *t*) (blue) and phase velocity *c*(*z* – *vt*) (orange). (a) Superluminal copropagating: modulation velocity *v* = 2*c*_b_, initial modulation position *Z*_1_/*Z*_p_ = −15 and *Z*_2_/*Z*_p_ = −5, snapshot times: *t*/*T*_p_ = 0, 5, 15, 25, 30. (b) Subluminal copropagating: modulation velocity *v* = *c*_b_/2, initial modulation position *Z*_1_/*Z*_p_ = 5 and *Z*_2_/*Z*_p_ = 15, snapshot times: *t*/*T*_p_ = 0,18,25,30,40. (c) Subluminal counterpropagating: modulation velocity *v* = −*c*_b_/2, initial modulation position *Z*_1_/*Z*_p_ = 20 and *Z*_2_/*Z*_p_ = 30, snapshot times: *t*/*T*_p_ = 0, 15.5, 17.5, 19, 22. (d) Superluminal counterpropagating: modulation velocity *v* = −2*c*_b_, initial modulation position *Z*_1_/*Z*_p_ = 20 and *Z*_2_/*Z*_p_ = 30, snapshot times: *t*/*T*_p_ = 0, 7, 8.5, 10.5, 12. The following set of parameters: *f*_1_ = 1, *f*_2_ = 1.5, *Z*_2_ – *Z*_1_ = 10*Z*_p_, Δ*z* = 1.25*Z*_p_, *T*_p_ = *Z*_p_/*c*_b_ were used in all four regimes. Videos associated with these snapshots are included as Supporting Information.

**Figure 4:**
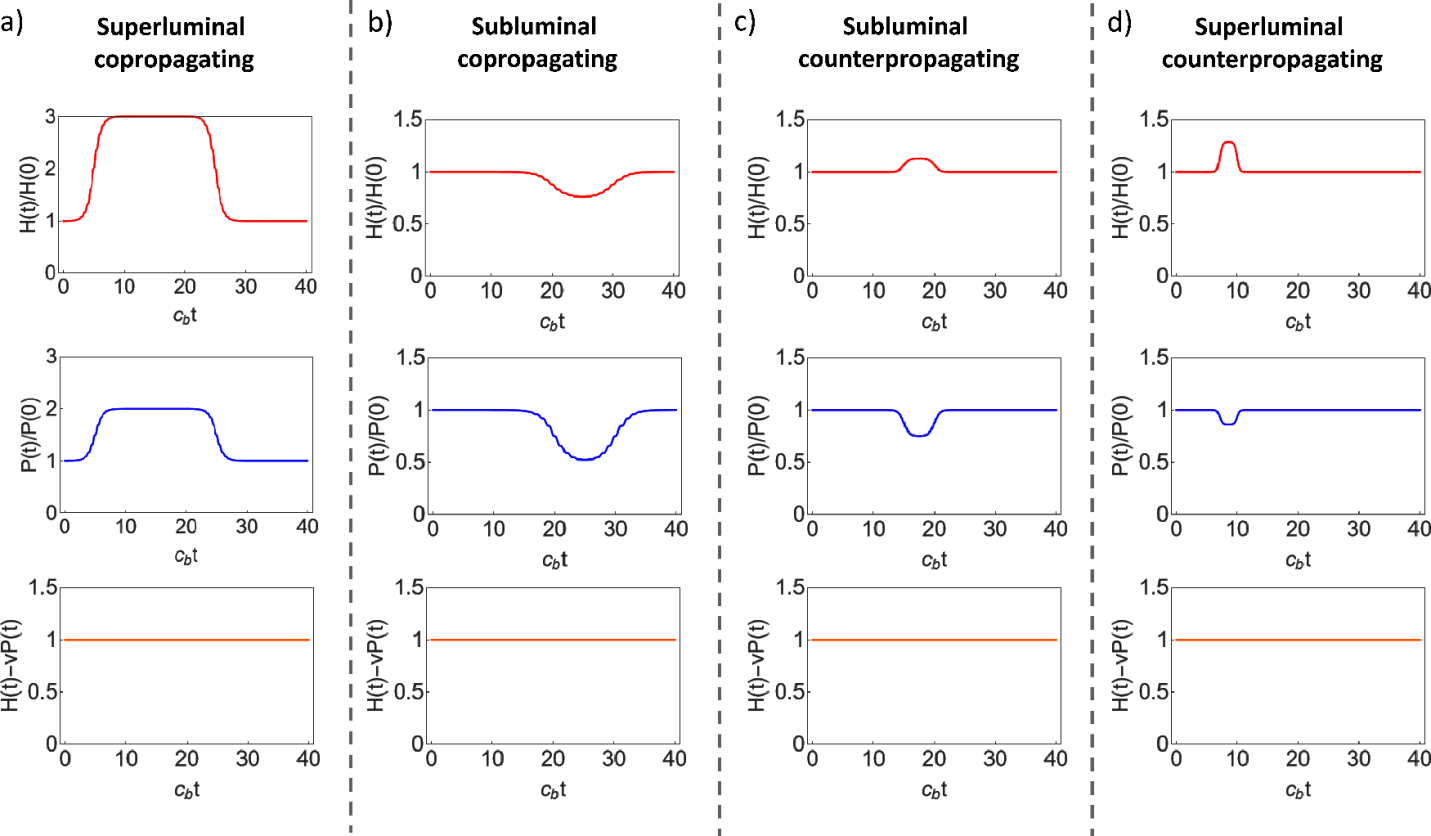
Time evolution of the energy, momentum and energy-momentum. Normalized energy *H*(*t*)/*H*(0), momentum *P*(*t*)/*P*(0), and energy-momentum [*H*(*t*) – *vP*(*t*)]/[*H*(0) – *vP*(0)], as a function of time, for three different regimes: (a) superluminal copropagating (*v* = 2*c*_b_), (b) subluminal copropagating (*v* = *c*_b_/2), (c) subluminal counterpropagating (*v* = −*c*_b_/2) and (d) superluminal counter propagating (*v* = −2*c*_b_). All simulation parameters are identical to those of Figure 3. The calculations confirm that both energy and momentum change in time, while the energy-momentum remains a conserved quantity.

Our choice of examples target four different regimes: (i) Superluminal copropagation (*v* = 2*c*_b_), (ii) subluminal copropagation (*v* = *c*_b_/2), (iii) subluminal counterpropagation (*v* = −*c*_b_/2), and (iv) superluminal counterpropagation in (*v* = −2*c*_b_). The results are gathered together in Figures 3 and 4. In addition, videos of the time evolution of the interactions for each example are included in Supporting Information. The following set of parameters: *f*_1_ = 1, *f*_2_ = 1.5, *Z*_2_ – *Z*_1_ = 10*Z*_p_, Δ*z* = 1.25*Z*_p_ (*Z*_p_ = 1) were used for all calculations.

First, in the case of superluminal copropagation (*v* = 2*c*_b_), the modulation pulse moves faster than the electromagnetic pulse, and the interaction takes place as the modulation passes through the electromagnetic pulse, which results in its compression (see Figure 3a). In this case, both the energy and momentum of the electromagnetic pulse are increased, while the energy-momentum is confirmed to be conserved (see Figure 4a). Moreover, the changes in energy are more prominent than those of the momentum, as expected from the superluminal regime. As *v* gets close the luminal regime, the overlapping time between the electromagnetic and modulation pulses increases, and the increase of energy and momentum are more pronounced (see Figure S1).

A qualitatively different scenario is observed in the case of subluminal copropagation (*v* = *c*_b_/2). For this configuration, the modulation is slower than the electromagnetic pulse, and the interaction takes place as the electromagnetic pulse passes through the modulation (see Figure 3b). Another difference is that the mode becomes expanded instead of being compressed. In other words, the pulse stretches as its front part enters into a spatiotemporal region with a higher speed of light. Moreover, the interaction with the modulation results in a decrease of both energy and momentum, while the energy-momentum is conserved (see Figure 4b). Our results also confirm that the changes in the momentum become dominant as the modulation velocity is decreased. In fact, in the exact zero velocity case (*v* = 0), the modulation becomes purely spatial and energy is conserved while the momentum is not (see Figure S2).

Yet a different regime appears in the case of subluminal counter-propagation (*v* = −*c*_b_/2). Within this regime, the electromagnetic pulse and the modulation collision against each other (see Figure 3c), resulting in an increase of the energy but a reduction of the momentum (see Figure 4c). A similar behavior is observed for the case of superluminal counterpropagation (*v* = 2*c*_b_), where the main difference is that the interaction takes place in a much shorter time scale (see Figure 3d), and it becomes more energy-like, as predicted by the conservation of the energy-momentum at high modulation speeds (see Figure 4d). Therefore, the transition from subluminal to superluminal does not present a qualitative change in the interaction, by contrast with the copropagating case. From a mathematical standpoint, this difference can be justified by the fact that *c*(*z* – *vt*) – *v* in eq 12 does not change sign in the counter-propagating case. In the deeply superluminal regime, the interaction results in a short impulse of energy, while the momentum remains constant, in agreement with the convergence with pure time-varying media (see Figure S3). To finalize, we remark that all the features observed in this section are consistent with the constraints discussed in the previous sections. In all examples it is found that the energy and momentum alternate between two constant values, corresponding to the regimes where either electromagnetic pulse and modulation do not overlap or they completely overlap. Moreover, the transient time between both regimes scales inversely with respect to the velocity difference |*v* – *c*_b_|, being the longest for copropagating luminal regimes, and the smallest for counterpropagating superluminal regimes. Furthermore, we note that while we used a box modulation with instantaneous boundaries, modulations with extended boundaries only result in an increase of the transition time for this impedance-matched case (see Figure S4).

As a final note, we remark that this work has focused on transversal fields propagating in parallel to the direction of the modulation for the sake of clarity a mathematical simplicity. Nevertheless, the conservation of the energy-momentum can be generalized to arbitrary fields and directions of modulation, as shown in Supporting Information Section 4 with the use of continuity equations. Specifically, it is demonstrated that the energy-momentum *H* – **v · P** is conserved in systems exhibiting with uniform velocity vector **v** = v **u**_v_, i.e., those characterized with permittivity ε(**r**, *t*) = ε(**r · u**_v_ – *t*) and permeability μ(**r**, *t*) = μ(**r · u**_v_ – *t*), which satisfy the conditions: ∂_*t*_ε(**r**, *t*) + **v · ∇** ε(**r**, *t*) = 0 and ∂_*t*_μ(**r**, *t*) + **v · ∇** μ(**r**, *t*) = 0.

## 8 Conclusions

Our analysis highlights that uniform spacetime metamaterials exhibit a spatiotemporal translation symmetry with a characteristic velocity defined by the velocity of the modulation. Following Noether’s theorem, the associated conserved quantity is an energy-momentum, i.e., a linear combination of the energy and Minkowski momentum, weighted by the velocity of the modulation. Moreover, the conservation of the energy-momentum imposes some constraints on the range of light-matter interactions that can take place within uniform spacetime metamaterials. Specifically, changes of energy and momentum are fundamentally intertwined by the modulation velocity. We expect that understanding these constraints will provide better design guidelines for the engineering of different light-matter interaction regimes in the realm of uniform spacetime metamaterials, and will motivate research on more general spacetime metamaterials that overcome them.

In general, our results highlight the existence of reduced symmetries and conserved quantities in spatiotemporal metamaterials, and the need to account for them in order to fully understand the range of allowed interactions. In other words, spacetime metamaterials break both spatial and temporal symmetries, but there are more generalized symmetries that should be considered. We expect that our research will inspire further research in this direction within the context of spacetime metamaterials. Finally, our results also broadly highlight the role of Noether’s theorem in understanding light-matter interactions in photonics, which might foster its applicability in related fields.

## Supporting Information Available

Animation of superluminal copropagating (AVI)
Animation of subluminal copropagating (AVI)
Animation of subluminal counterpropagating (AVI)
Animation of superluminal counterpropagating (AVI)
Theoretical derivations, supplementary figures with additional examples, supplementary videos with animations of the examples provided in Figure 3, and generalization of the energy-momentum conservation for arbitrary fields and modulation velocity directions (PDF)

## Supplementary Material

ph4c01496_si_001.avi

ph4c01496_si_002.avi

ph4c01496_si_003.avi

ph4c01496_si_004.avi
