# Supplementary material for: Spatiotemporal Symmetries and Energy-Momentum Conservation in Uniform Spacetime Metamaterials
Source: ACS Photonics. 2024 Nov 13;11(12):5273–80. doi: 10.1021/acsphotonics.4c01496 (PMC11660235; doi:10.1021/acsphotonics.4c01496)
Supplement: Supplementary file 5 — ph4c01496_si_005.pdf [file ph4c01496_si_005.pdf]

Supplementary Information for: "Spatiotemporal symmetries and energy-momentum conservation in uniform spacetime metamaterials"

Iñigo Liberal, Antonio Ganfornina-Andrades and J. Enrique Vázquez-Lozano

Department of Electrical, Electronic and Communications Engineering, Institute of Smart Cities (ISC), Public University of Navarre (UPNA), 31006 Pamplona, Spain. Inigo.liberal@unavarra.es

This document includes:

1. Supplementary Information Section 1-4

2. Supplementary Figures S1-S4

Supplementary Information for "Spatiotemporal symmetries and energy-momentum conservation in uniform spacetime metamaterials"

Iñigo Liberal, Antonio Ganfornina-Andrades and J. Enrique Vázquez-Lozano

October 31, 2024

1 Symmetry of the wave equation: Derivation of Eq. (3)

This Supplementary Information section provides additional details on the derivation of Eq. (3) of the main text, i.e., the wave equation satisfied by the transformed vector potential. Our starting point is the wave equation in space-time modulated media, i.e., Eq. (1) of the main text:

$$\partial_z \left\{\mu^{-1}(z,t)\,\partial_z A_x(z,t)\right\} = \partial_t \left\{\varepsilon(z,t)\,\partial_t A_x(z,t)\right\} \tag{1}$$

It will be convenient to re-arrange it in the following manner

$$\partial_z^2 A_x(z,t) = \mu(z,t)\,\varepsilon(z,t)\,\partial_t^2 A_x(z,t) + \mu(z,t)\,\partial_t\varepsilon(z,t)\,\partial_t A_x(z,t) - \partial_z\mu^{-1}(z,t)\,\partial_z A_x(z,t) \tag{2}$$

Then, we define a continuous spatiotemporal translation with characteristic velocity v:

$$A_x'(z,t) = A_x(z,t) + dA_x(z,t) \tag{3}$$

with

$$dA_x(z,t) = dt\,\left(\partial_t + v\partial_z\right) A_x(z,t) \tag{4}$$

By taking the second spatial derivative in (3) and introducing (4) we find:

$$\partial_z^2 A_x'(z,t) = \partial_z^2 A_x(z,t) + dt\,\left(\partial_t + v\partial_z\right)\partial_z^2 A_x(z,t) \tag{5}$$

Then, we introduce (2) to find:

$$\partial_z^2 A'_x(z,t) = \mu(z,t)\,\varepsilon(z,t)\,\partial_t^2 A_x(z,t) + \mu(z,t)\,\partial_t\varepsilon(z,t)\,\partial_t A_x(z,t) - \partial_z\mu^{-1}(z,t)\,\partial_z A_x(z,t)$$

$$+dt\;(\partial_t + v\partial_z)\left\{\mu(z,t)\,\varepsilon(z,t)\,\partial_t^2 A_x(z,t) + \mu(z,t)\,\partial_t\varepsilon(z,t)\,\partial_t A_x(z,t) - \partial_z\mu^{-1}(z,t)\,\partial_z A_x(z,t)\right\} \tag{6}$$

$$= \mu(z,t)\,\varepsilon(z,t)\,\partial_t^2 A_x(z,t) + \mu(z,t)\,\partial_t\varepsilon(z,t)\,\partial_t A_x(z,t) - \partial_z\mu^{-1}(z,t)\,\partial_z A_x(z,t)$$

$$+\mu(z,t)\,\varepsilon(z,t)\,\partial_t^2\left\{dt\;(\partial_t + v\partial_z)\,A_x(z,t)\right\}$$

$$+\mu(z,t)\,\partial_t\varepsilon(z,t)\,\partial_t\left\{dt\;(\partial_t + v\partial_z)\,A_x(z,t)\right\}$$

$$-\partial_z\mu^{-1}(z,t)\,\partial_z\left\{dt\;(\partial_t + v\partial_z)\,A_x(z,t)\right\}$$

$$+dt\;(\partial_t + v\partial_z)\left\{\mu(z,t)\,\varepsilon(z,t)\right\}\partial_t^2 A_x(z,t)$$

$$+dt\;(\partial_t + v\partial_z)\left\{\mu(z,t)\,\partial_t\varepsilon(z,t)\right\}\partial_t A_x(z,t)$$

$$-dt\;(\partial_t + v\partial_z)\left\{\partial_z\mu^{-1}(z,t)\right\}\partial_z A_x(z,t) \tag{7}$$

$$= \mu(z,t)\,\varepsilon(z,t)\,\partial_t^2 A'_x(z,t) + \mu(z,t)\,\partial_t\varepsilon(z,t)\,\partial_t A'_x(z,t) - \partial_z\mu^{-1}(z,t)\,\partial_z A'_x(z,t)$$

$$+dt\;(\partial_t + v\partial_z)\left\{\mu(z,t)\,\varepsilon(z,t)\right\}\partial_t^2 A_x(z,t)$$

$$+dt\;(\partial_t + v\partial_z)\left\{\mu(z,t)\,\partial_t\varepsilon(z,t)\right\}\partial_t A_x(z,t)$$

$$-dt\;(\partial_t + v\partial_z)\left\{\partial_z\mu^{-1}(z,t)\right\}\partial_z A_x(z,t) \tag{8}$$

which is equivalent to Eq. (3) from the main text.

2 Continuity equations: Derivation of Eqs. (9)-(12) of the main text.

This Supplementary Information section provides additional details on the derivation of Eqs. (9)-(12) of the main text, i.e., the continuity equations for energy, momentum and energy-momentum in spacetime metamaterials. To this end, we start with the definition of the energy density, expressed in terms of the vector potential $A_x(z,t)$:

$$h(z,t) = \frac{\varepsilon(z,t)}{2}\left(\partial_t A_x(z,t)\right)^2 + \frac{\mu^{-1}(z,t)}{2}\left(\partial_z A_x(z,t)\right)^2 \tag{9}$$

First, we take the time derivative

$$\begin{aligned}\partial_t h(z,t) &= \frac{\partial_t \varepsilon(z,t)}{2}\left(\partial_t A_x(z,t)\right)^2 + \frac{\varepsilon(z,t)}{2}\partial_t\left(\partial_t A_x(z,t)\right)^2 + \frac{1}{2}\partial_t \mu^{-1}(z,t)\left(\partial_z A_x(z,t)\right)^2 + \frac{\mu^{-1}(z,t)}{2}\partial_t\left(\partial_z A_x(z,t)\right)^2\\ &= \frac{\partial_t \varepsilon(z,t)}{2}\left(\partial_t A_x(z,t)\right)^2 + \frac{1}{2}\partial_t \mu^{-1}(z,t)\left(\partial_z A_x(z,t)\right)^2\\ &+\varepsilon(z,t)\,\partial_t^2 A_x(z,t) + \mu^{-1}(z,t)\,\partial_z A_x(z,t)\,\partial_t\partial_z A_x(z,t)\end{aligned} \tag{10}$$

Next, we rearrange Eq. (1) to write

$$\varepsilon(z,t)\,\partial_t^2 A_x(z,t) = \partial_z\left\{\mu^{-1}(z,t)\,\partial_z A_x(z,t)\right\} - \partial_t \varepsilon(z,t)\,\partial_t A_x(z,t) \tag{11}$$

Leading to

$$\begin{aligned}\partial_t h(z,t) &= \frac{\partial_t \varepsilon(z,t)}{2}\left(\partial_t A_x(z,t)\right)^2 + \frac{\partial_t \mu^{-1}(z,t)}{2}\left(\partial_z A_x(z,t)\right)^2\\ &+\partial_t A_x(z,t)\,\partial_z\left\{\mu^{-1}(z,t)\,\partial_z A_x(z,t)\right\} - \partial_t \varepsilon(z,t)\left(\partial_t A_x(z,t)\right)^2 + \mu^{-1}(z,t)\,\partial_z A_x(z,t)\,\partial_t\partial_z A_x(z,t)\end{aligned} \tag{12}$$

$$\begin{aligned}&= -\frac{\partial_t \varepsilon(z,t)}{2}\left(\partial_t A_x(z,t)\right)^2 + \frac{\partial_t \mu^{-1}(z,t)}{2}\left(\partial_z A_x(z,t)\right)^2\\ &+\partial_z\left\{\mu^{-1}(z,t)\,\partial_t A_x(z,t)\,\partial_z A_x(z,t)\right\}\end{aligned} \tag{13}$$

So that we can compactly write

$$\partial_t h(z,t) + \partial_z F_h(z,t) = J_h(z,t) \tag{14}$$

with

$$F_h(z,t) = -\mu^{-1}(z,t)\ \partial_t A_x(z,t)\,\partial_z A_x(z,t) \tag{15}$$

and

$$J_h(z,t) = -\frac{\partial_t \varepsilon(z,t)}{2}\ (\partial_t A_x(z,t))^2 + \frac{\partial_t \mu^{-1}(z,t)}{2}\ (\partial_z A_x(z,t))^2 \tag{16}$$

Similarly, we define the Minkowski momentum density

$$p(z,t) = -\varepsilon(z,t)\,\partial_t A_x(z,t)\,\partial_z A_x(z,t) \tag{17}$$

Then, we take the time derivative

$$\partial_t p(z,t) = -\partial_t \varepsilon(z,t)\,\partial_t A_x(z,t)\,\partial_z A_x(z,t) - \varepsilon(z,t)\,\partial_t^2 A_x(z,t)\,\partial_z A_x(z,t) - \varepsilon(z,t)\,\partial_t A_x(z,t)\,\partial_z \partial_t A_x(z,t) \tag{18}$$

Using again (11) we find

$$\partial_t p(z,t) = -\partial_z \mu^{-1}(z,t)\,(\partial_z A_x(z,t))^2 - \varepsilon(z,t)\,\partial_t A_x(z,t)\,\partial_z \partial_t A_x(z,t) - \mu^{-1}(z,t)\,\partial_z A_x(z,t)\,\partial_z^2 A_x(z,t) \tag{19}$$

Then, we note the following relation

$$\begin{aligned}
&\frac{1}{2}\partial_z\left\{\varepsilon(z,t)\,(\partial_t A_x(z,t))^2 + \mu^{-1}(z,t)\,(\partial_z A_x(z,t))^2\right\} \\
&= \frac{1}{2}\partial_z \varepsilon(z,t)\,(\partial_t A_x(z,t))^2 + \frac{1}{2}\partial_z \mu^{-1}(z,t)\,(\partial_z A_x(z,t))^2 \\
&+\varepsilon(z,t)\,\partial_t A_x(z,t)\,\partial_z \partial_t A_x(z,t) + \mu^{-1}(z,t)\,\partial_z A_x(z,t)\,\partial_z^2 A_x(z,t)
\end{aligned} \tag{20}$$

So that we can write

$$\partial_t p(z,t) + \partial_z F_p(z,t) = J_p(z,t) \tag{21}$$

with

$$F_p\left(z,t\right)=\frac{1}{2}\,\varepsilon\left(z,t\right)\left(\partial_t A_x\left(z,t\right)\right)^2+\mu^{-1}\left(z,t\right)\left(\partial_z A_x\left(z,t\right)\right)^2 \tag{22}$$

and

$$J_p\left(z,t\right)=\frac{1}{2}\partial_z\varepsilon\left(z,t\right)\left(\partial_t A_x\left(z,t\right)\right)^2-\frac{1}{2}\partial_z\mu^{-1}\left(z,t\right)\left(\partial_z A_x\left(z,t\right)\right)^2 \tag{23}$$

Finally, combining the continuity equations for the energy and momentum we can find the continuity equation for the energy-momentum:

$$\partial_t\left\{h\left(z,t\right)-vp\left(z,t\right)\right\}+\partial_z\left\{F_h\left(z,t\right)-vF_p\left(z,t\right)\right\}=$$

$$=-\frac{1}{2}\left\{\left(\partial_t+v\partial_z\right)\left\{\varepsilon\left(z,t\right)\right\}\left(\partial_t A_x\left(z,t\right)\right)^2-\left(\partial_t+v\partial_z\right)\left\{\mu^{-1}\left(z,t\right)\right\}\left(\partial_z A_x\left(z,t\right)\right)^2\right\} \tag{24}$$

3 Method of charateristics

In this Supplementary Information section we provide additional details on the theoretical methods employed to solve the numerical examples reported in Figs. 3 and 4 of the main text.

First, we note that the examples focus on impedance-matched modulations: $\varepsilon(z,t) = \varepsilon_b f(z - vt)$ and $\mu(z,t) = \mu_b f(z - vt)$, such that $Z(z,t) = \sqrt{\frac{\mu(z,t)}{\varepsilon(z,t)}} = \sqrt{\frac{\mu_b}{\varepsilon_b}} = Z_b$. On the other hand, the phase velocity reduces to $c(z,t) = 1/\sqrt{\mu(z,t)\,\varepsilon(z,t)} = c_b/f(z - vt)$. For this impedance-matched modulations no backward waves are excited. Thus, for waves propagating along $+\widehat{\mathbf{z}}$ we can write: $E_x(z,t)/H_y(z,t) = Z_b$.

Using this property, we can rewrite Maxwell equations as a first-order partial differential equation (PDE) for the displacement field

$$\partial_z H_y(z,t) = -\partial_t D_x(z,t) \rightarrow \partial_z \{c(z - vt)\, D_x(z,t)\} + \partial_t D_x(z,t) = 0 \tag{25}$$

Therefore, the scattering problem has been reduced to solving a PDE with some given initial conditions $D_x(z_0, 0) = F(z_0)$ at $t = 0$ before the interactions. We solve this PDE by converting it into a ordinary differential equation (ODE) via the method of characteristics. To this end, first we make the change of variables $Z = z - vt$ and $T = t$, so that Eq. (25) can be rewritten as follows

$$\partial_Z \{[c(Z) - v]\, D_x(Z,T)\} + \partial_T D_x(Z,T) = 0 \tag{26}$$

Next, we define an auxiliary field

$$\psi(Z,T) = [c(Z) - v]\, D_x(Z,T) \tag{27}$$

which obeys the PDE:

$$\partial_Z \psi(Z,T) + \frac{1}{c(Z) - v}\,\partial_T \psi(Z,T) = 0 \tag{28}$$

This PDE can be conveniently solved via the method of characteristics. To this end, we want to identify the trayectories $T(Z)$ where $\psi(Z,T)$ does not change:

$$\frac{d}{dZ}\{\psi(Z,T(Z))\} = \frac{\partial\psi}{\partial Z} + \frac{\partial\psi}{\partial T}\frac{\partial T}{\partial Z} = 0 \tag{29}$$

And we identify that this is indeed a solution of our equation along the trayectory defined by

$$\frac{dT}{dZ} = \frac{1}{c(Z) - v} \tag{30}$$

with solution

$$T(Z) = \int_{Z_c}^{Z} dZ' \frac{1}{c(Z') - v} + T_c \tag{31}$$

where $T_c = T(Z_c)$ is the crossing of the trayectories on the axis $Z = Z_c$, which characterizes each different trayectory. Along these trayectories $\psi(Z, T(Z)) = \textit{constant}$ is a constant that can be linked to the initial conditions: $D_x(z_0, 0) = F(z_0)$. To this end we note that $Z(z, 0) = z$ and $T(z, 0) = 0$. In other words, the $t = 0$ axis in the original coordinate systems directly corresponds to the $T = 0$ axis in the transformed coordinate system. Therefore, the intial values $D_x(z_0, 0) = F(z_0)$ can be directly asigned to the cuts of $\psi(Z, T(Z))$ on the $T = 0$ axis.

In other words, suppose that we want to evaluate $D_x(z, t)$ at a given point (z, t). First, we identify that this corresponds to the transformed coordinates: $(Z = z - vt, T = t)$. Second, we identify in which trayectory $\{Z, T(Z)\}$, characterized with parameter T_c, this point lies, which can be done as follows

$$T_c = T - \int_{Z_c}^{Z} dZ' \frac{1}{c(Z') - v} \tag{32}$$

Then, we need to find the initial point associated with such trayectory, which corresponds to the Z_0 value where the transformed time is zero, leading to the equation

$$T_c + \int_{Z_c}^{Z_0} dZ' \frac{1}{c(Z') - v} = 0 \tag{33}$$

After solving this equation, we can then associate $\psi(Z, T(Z)) = \psi(Z_0, 0)$. Finally, the electric field displacement is given by $D_x(x, t) = \psi(Z_0, 0) / [c(z - vt) - v]$, so that

$$D_x(x, t) = \frac{c(Z_0) - v}{c(z - vt) - v} D_x(Z_0, 0) \tag{34}$$

which corresponds to Eq. (12) of the main text.

Next, we focus on the particular case of a step modulation moving at uniform speed, i.e., the spatiotemporal modulation employed in the examples of Fig. 3 and 4. Then, the phase velocity is given by

$$c(z - vt) = \begin{cases} c_1 & z - vt \leq Z_1 \\ c_2 & Z_1 < z - vt < Z_2 \\ c_1 & z - vt \geq Z_2 \end{cases} \tag{35}$$

Consequently, the trayectories can be explicitly written as

$$
\begin{aligned}
T(Z) &= \int_{Z_c}^{Z} dZ' \, \frac{1}{c(Z') - v} + T_c \\
&= \begin{cases} T_c + \frac{Z - Z_c}{c_1 - v} & Z \leq Z_1 \\ T_c + \frac{Z_1 - Z_c}{c_1 - v} + \frac{Z - Z_1}{c_2 - v} & Z_1 < Z < Z_2 \\ T_c + \frac{Z_1 - Z_c}{c_1 - v} + \frac{Z_2 - Z_1}{c_2 - v} + \frac{Z - Z_2}{c_1 - v} & Z \geq Z_2 \end{cases}
\end{aligned} \tag{36}
$$

And Z_0 must satisfy (33) with solution

$$
Z_0 = \begin{cases} -(c_1 - v)\, T_c + Z_c & -(c_1 - v)\, T_c \leq Z_1 \\ -(c_2 - v)\left[T_c + \frac{Z_1 - Z_c}{c_1 - v}\right] + Z_1 & Z_1 < -(c_2 - v)\left[T_c + \frac{Z_1 - Z_c}{c_1 - v}\right] + Z_1 < Z_2 \\ -(c_1 - v)\left[T_c + \frac{Z_1 - Z_c}{c_1 - v} + \frac{Z_2 - Z_1}{c_2 - v}\right] + Z_2 & -(c_1 - v)\left[T_c + \frac{Z_1 - Z_c}{c_1 - v} + \frac{Z_2 - Z_1}{c_2 - v}\right] + Z_2 \geq Z_2 \end{cases} \tag{37}
$$

4 Generalization to arbitrary fields and modulation directions

In this Supplementary Information section we generalize the conservation of the energy-momentum to arbitrary fields and modulation directions by using continuity equations. To this end, we consider a uniform spacetime metamaterial with modulation velocity vector $\mathbf{v} = v\,\mathbf{u}_v$, characterized by permittivity and permeability

$$\varepsilon(\mathbf{r},t) = \varepsilon(\mathbf{r}\cdot\mathbf{u}_v - vt) \tag{38}$$

$$\mu(\mathbf{r},t) = \mu(\mathbf{r}\cdot\mathbf{u}_v - vt) \tag{39}$$

which satisfy the conditions:

$$\partial_t\varepsilon(\mathbf{r},t) + \mathbf{v}\cdot\nabla\varepsilon(\mathbf{r},t) = 0 \tag{40}$$

$$\partial_t\mu(\mathbf{r},t) + \mathbf{v}\cdot\nabla\mu(\mathbf{r},t) = 0 \tag{41}$$

Next, taking the time-derivative of the energy density we find

$$\begin{aligned}
\partial_t h &= \frac{1}{2\varepsilon(\mathbf{r},t)}\partial_t\mathbf{D}^2 + \frac{1}{2\mu(\mathbf{r},t)}\partial_t\mathbf{B}^2 + \frac{1}{2}\partial_t\left\{\frac{1}{\varepsilon(\mathbf{r},t)}\right\}\mathbf{D}^2 + \frac{1}{2}\partial_t\left\{\frac{1}{\mu(\mathbf{r},t)}\right\}\mathbf{B}^2\\
&= \frac{1}{\varepsilon(\mathbf{r},t)}\mathbf{D}\cdot\partial_t\mathbf{D} + \frac{1}{\mu(\mathbf{r},t)}\mathbf{B}\cdot\partial_t\mathbf{B} - \frac{1}{2}\frac{1}{\varepsilon^2(\mathbf{r},t)}\partial_t\varepsilon\mathbf{D}^2 - \frac{1}{2}\frac{1}{\mu^2(\mathbf{r},t)}\partial_t\mu\mathbf{B}^2\\
&= \mathbf{E}\cdot\nabla\times\mathbf{H} - \mathbf{H}\cdot\nabla\times\mathbf{E} - \frac{1}{2}\partial_t\varepsilon(\mathbf{r},t)\,\mathbf{E}^2 - \frac{1}{2}\partial_t\mu(\mathbf{r},t)\,\mathbf{H}^2\\
&= -\nabla\cdot(\mathbf{E}\times\mathbf{H}) - \frac{1}{2}\partial_t\varepsilon(\mathbf{r},t)\,\mathbf{E}^2 - \frac{1}{2}\partial_t\mu(\mathbf{r},t)\,\mathbf{H}^2
\end{aligned} \tag{42}$$

Leading to the continuity equation

$$\partial_t h + \nabla\cdot\mathbf{S} = -\frac{1}{2}\partial_t\varepsilon(\mathbf{r},t)\,\mathbf{E}^2 - \frac{1}{2}\partial_t\mu(\mathbf{r},t)\,\mathbf{H}^2 \tag{43}$$

Similarly, taking the time-derivative of the momentum density we find

$$\partial_t\mathbf{p} = \partial_t(\mathbf{D}\times\mathbf{B}) = \partial_t\mathbf{D}\times\mathbf{B} + \mathbf{D}\times\partial_t\mathbf{B} =$$

$$= \varepsilon(\mathbf{r},t)(\nabla\times\mathbf{E})\times\mathbf{E} + \mu(\mathbf{r},t)(\nabla\times\mathbf{H})\times\mathbf{H}$$

$$= \varepsilon(\mathbf{r},t)(\mathbf{E}\cdot\nabla)\mathbf{E} - \frac{1}{2}\varepsilon(\mathbf{r},t)\nabla\mathbf{E}^2 + \mu(\mathbf{r},t)(\mathbf{H}\cdot\nabla)\mathbf{H} - \frac{1}{2}\mu(\mathbf{r},t)\nabla\mathbf{H}^2$$

$$= \varepsilon(\mathbf{r},t)(\mathbf{E}\cdot\nabla)\mathbf{E} - \frac{1}{2}\nabla\left(\varepsilon(\mathbf{r},t)\mathbf{E}^2\right) + \frac{1}{2}\nabla\varepsilon(\mathbf{r},t)\mathbf{H}^2 + \mu(\mathbf{r},t)(\mathbf{H}\cdot\nabla)\mathbf{H} - \frac{1}{2}\nabla\left(\mu(\mathbf{r},t)\mathbf{H}^2\right) + \frac{1}{2}\nabla\mu(\mathbf{r},t)\mathbf{H}^2$$

$$= \nabla\cdot\left(\varepsilon(\mathbf{r},t)\mathbf{E}\mathbf{E} - \frac{1}{2}\overline{\overline{\mathbf{I}}}\varepsilon(\mathbf{r},t)\mathbf{E}^2\right) + \frac{1}{2}\nabla\varepsilon(\mathbf{r},t)\mathbf{E}^2 + \nabla\cdot\left(\mu(\mathbf{r},t)\mathbf{H}\mathbf{H} - \frac{1}{2}\overline{\overline{\mathbf{I}}}\mu(\mathbf{r},t)\mathbf{H}^2\right) + \frac{1}{2}\nabla\mu(\mathbf{r},t)\mathbf{H}^2 \quad (44)$$

Leading to the continuity equation

$$\partial_t\mathbf{p} + \nabla\cdot\overline{\overline{\mathbf{T}}} = \frac{1}{2}\nabla\varepsilon(\mathbf{r},t)\mathbf{E}^2 + \frac{1}{2}\nabla\mu(\mathbf{r},t)\mathbf{H}^2 \quad (45)$$

Combining both continuity equations we find that the time-evolution of the energy-momentum is given by

$$\partial_t\{h - \mathbf{v}\cdot\mathbf{p}\} + \nabla\cdot\mathbf{S} - \mathbf{v}\cdot\nabla\cdot\overline{\overline{\mathbf{T}}}_M =$$

$$= -\frac{1}{2}\{\partial_t\varepsilon(\mathbf{r},t) + \mathbf{v}\cdot\nabla\varepsilon(\mathbf{r},t)\}\mathbf{E}^2 - \frac{1}{2}\{\partial_t\mu(\mathbf{r},t) + \mathbf{v}\cdot\nabla\mu(\mathbf{r},t)\}\mathbf{H}^2 \quad (46)$$

Finally, it can be concluded that the source/sink terms (46) are zero for spatiotemporal modulations satisfying the conditions (40)-(41). Therefore, the energy-momentum is conserved in uniform spacetime metamaterials, irrespectively of the field distribution and/or the direction of the modulation velocity.

Supplementary Figures:

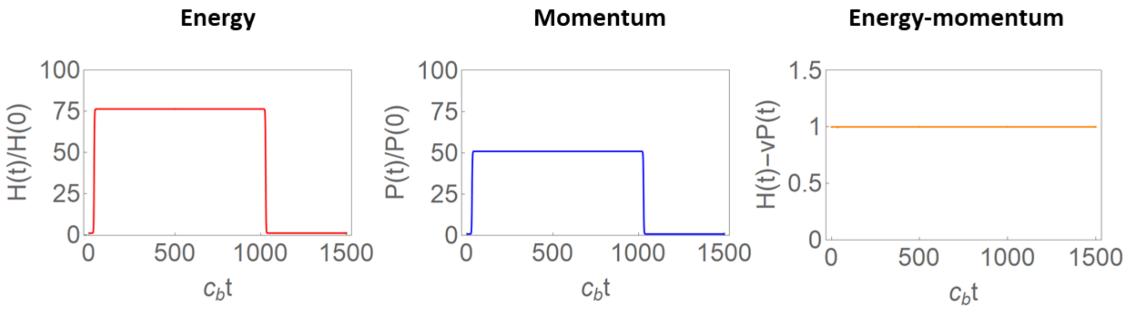

Fig. S1. Time evolution of the energy, momentum and energy-momentum in the near-luminal regime $v = 1.51c_b$. Normalized energy $H(t)/H(0)$, momentum $P(t)/P(0)$, and energy-momentum $[H(t) - vP(t)]/[H(0) - vP(0)]$. The following set of parameters: $Z_1/Z_p = -25$, $Z_2/Z_p = -15$, $f_1 = 1$, $f_2 = 1.5$, $Z_2 - Z_1 = 10Z_p$, $\Delta z = 1.25Z_p$, $T_p = Z_p/c_b$ were used in the calculations. The figure illustrates how in the near-luminal regime is characterized by large overlapping times and strong energy and momentum amplification.

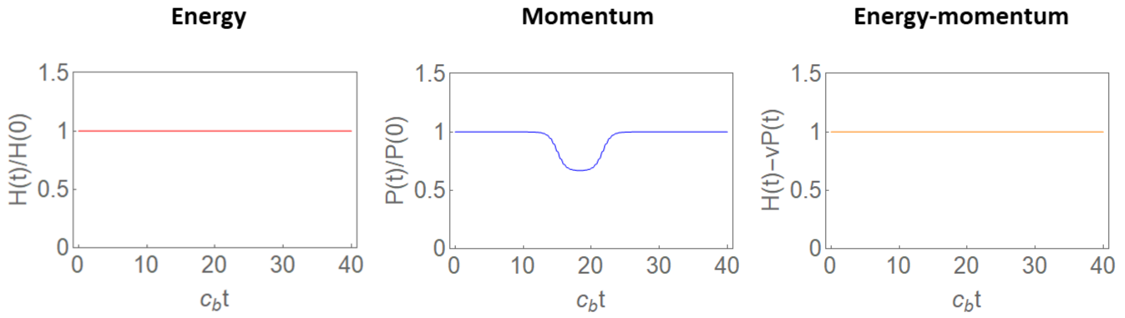

Fig. S2. Time evolution of the energy, momentum and energy-momentum in the zero-velocity regime $v = 0c_b$. Normalized energy $H(t)/H(0)$, momentum $P(t)/P(0)$, and energy-momentum $[H(t) - vP(t)]/[H(0) - vP(0)]$. The following set of parameters: $Z_1/Z_p = 15$, $Z_2/Z_p = 25$, $f_1 = 1$, $f_2 = 1.5$, $Z_2 - Z_1 = 10Z_p$, $\Delta z = 1.25Z_p$, $T_p = Z_p/c_b$ were used in the calculations. The figure illustrates how energy is conserved in the zero-velocity regime, while the momentum is not, and the energy-momentum converges to the energy.

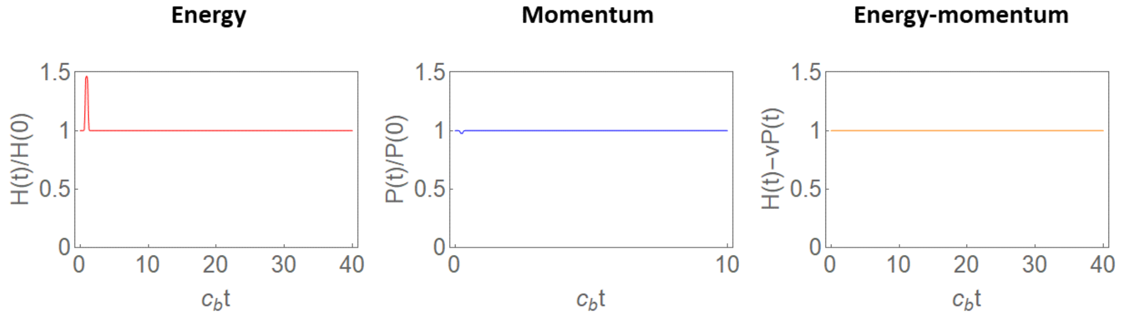

Fig. S3. Time evolution of the energy, momentum and energy-momentum in the deeply superluminal regime $v = -20c_b$. Normalized energy $H(t)/H(0)$, momentum $P(t)/P(0)$, and energy-momentum $[H(t) - vP(t)]/[H(0) - vP(0)]$. The following set of parameters: $Z_1/Z_p = 15$, $Z_2/Z_p = 25$, $f_1 = 1$, $f_2 = 1.5$, $Z_2 - Z_1 = 10Z_p$, $\Delta z = 1.25Z_p$, $T_p = Z_p/c_b$ were used in the calculations. The figure illustrates how in the deeply superluminal regime energy the interaction takes place in very short time-scales, leading to an energy bump while the momentum is conserved.

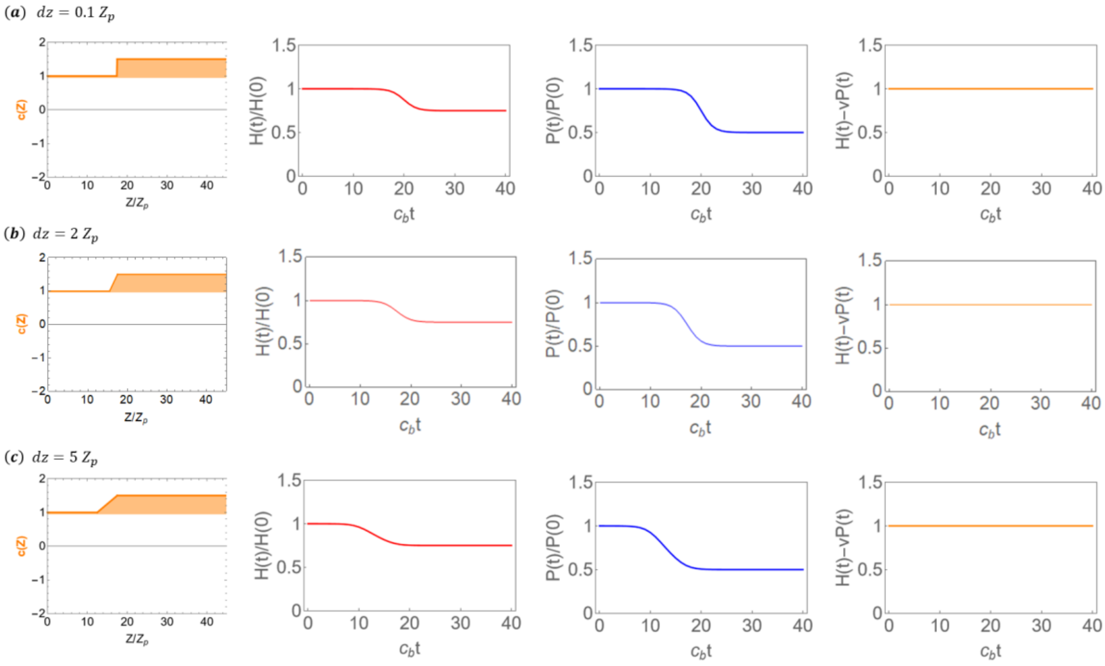

Fig. S4. Impact of the boundary width on the examples. Time evolution of the energy, momentum and energy-momentum for a moving boundary of linear profile with a width of (a) $dz = 0.1\ Z_p$, (b) $dz = 2\ Z_p$ and (c) (a) $dz = 5\ Z_p$, moving within the subluminal co-propagating regime ($v = c_b/2$). Normalized energy $H(t)/H(0)$, momentum $P(t)/P(0)$, and energy-momentum $[H(t) - vP(t)]/[H(0) - vP(0)]$. The following set of parameters: $Z_1/Z_p = 10$, $f_1 = 1$, $f_2 = 1.5$, $T_p = Z_p/c_b$ were used in the calculations. The figure illustrates how in in this impedance-matched example the width of the boundary only impacts the transition time.
